# Supplementary material for: How People in Eight European Countries Felt About the Safety, Effectiveness, and Necessity of COVID-19 Vaccination: A Cross-Sectional Survey
Source: Healthcare (Basel). 2025 Feb 6;13(3):344. doi: 10.3390/healthcare13030344 (PMC11817506; doi:10.3390/healthcare13030344)
Supplement: Supplementary file 1 [file healthcare-13-00344-s001.zip › healthcare-3345432-supplementary.pdf]

Supplementary File S1. English version of the questionnaire.

Dear participant, we are researchers from five different countries, affiliated to the European General Practice Research Network (EGPRN). In our research, we focus on primary care and public health. We created this questionnaire together in order to learn about the views and ideas of people without a healthcare qualification about COVID-19 vaccination. By completing this questionnaire, you can help tailor vaccine information to the needs of the public. We would greatly appreciate your participation. It is an anonymous questionnaire, which takes about 10-15 minutes to complete. Thank you in advance for sharing your opinion!

About you

These questions help us to better understand the people who answered our survey. We use them to combine and compare your views and ideas with those of others.

1. Sex:
- Male
- Female
- Other
- Prefer not to say

2. Age: ____________________ years

3. Which country do you live in?
- Albania
- Belgium
- Croatia
- Germany
- Italy
- Latvia
- Macedonia
- Slovenia
- Another country

4. Do you consider yourself as part of a minority group(s) in the country that you live in?
- Yes
- No
- Prefer not to say
- Don't know

* You answered **Yes.** Which minority group(s) are you part of? _____________

5. Highest education level:

- Elementary school
- High school
- Bachelor's degree (or equivalent)
- Master's degree (or equivalent)
- Doctoral degree
- Prefer not to say

6. Area of living:
- Inner city
- Suburban
- Rural
- Other

7. Do you live alone?
- Yes
- No

8. Do you have children under 18 years old?
- Yes
- No

9. Employment:
- Working Full-Time
- Working Part-Time
- Studying full time
- Retired
- Unemployed and looking for a job
- Unemployed and unable to work
- Prefer not to say

10. Do you personally know anyone who has been hospitalized for COVID-19? (Choose all that apply)
- No one
- Myself
- A family member
- A friend
- A work colleague
- A neighbour
- Other

Your views on vaccines
We would like to know more about your views on vaccines and vaccination programs; both in general and specifically for COVID-19.

11. Have you had all the regular vaccines on the vaccination schedule in your country (for example: polio, tetanus, measles)?
- Yes
- No -> Q11a
- I don't know

11a. Why did you not have all vaccines?

____

COVID-19 vaccination

12. COVID-19 vaccines are safe.
Strongly agree - Agree - Neutral - Disagree - Strongly disagree

13. Why? ________________

14. COVID-19 vaccines are effective.
Strongly agree - Agree - Neutral - Disagree - Strongly disagree

15. Why? ________________

16. COVID-19 vaccines are necessary.
Strongly agree - Agree - Neutral - Disagree - Strongly disagree

17. Why? ________________

18. Have you been vaccinated* for COVID-19? (*Have had at least 1 dose)
- Yes - > go to question 20
- No -> go to question 19

19. Would you like to be vaccinated for COVID-19?
- Yes
- No

20. Why did you want/not want to be vaccinated?

____

21. What do you think are the advantages of having the COVID-19 vaccine?

____

22. What do you think are the disadvantages of having the COVID-19 vaccine?

____

23. If you could choose, which COVID-19 vaccine would you prefer?

- AstraZeneca (Vaxzevria)
- Covaxin (Bharat Biotech)
- Covishield (Serum Institute of India)
- Covovax (Serum Institute of India)
- Gamaleya (SputnikV)
- Janssen (Johnson & Johnson)
- Moderna (Spikevax)
- Nuvaxovid (Novavax)
- Pfizer-BioNTech (Comirnaty)
- Sinopharm
- Sinovac (CoronaVac)
- No preference

24. What would make you choose this vaccine?

25. In your country, have you had a choice of which COVID-19 vaccine you get?

- Yes
- No
- Mixed (for example: no for the first dose, but yes for second or third)

Your knowledge about vaccines; where you get information, and how much you trust that information

We would love to get your opinion on the COVID-19 vaccination program and informational services in your country.

26. I am well informed about the different COVID-19 vaccines used in my country's vaccination program.

Strongly disagree – disagree – neutral – agree – strongly agree

27. Did you receive information in your own language?

- Yes
- No

28. What questions about COVID-19 vaccines would you like more information on?

____

29. Who do you trust most to answer these questions?

30. Why do you trust this source the most?

31. What do you think about the COVID-19 vaccine information campaign in your country? Why?

32. What do you think is the best way to increase vaccination rates?

33. Which, if any, COVID-19 vaccination campaigns of other countries would you like to see in your own country? Why?

Supplementary File S2. Coding tree.

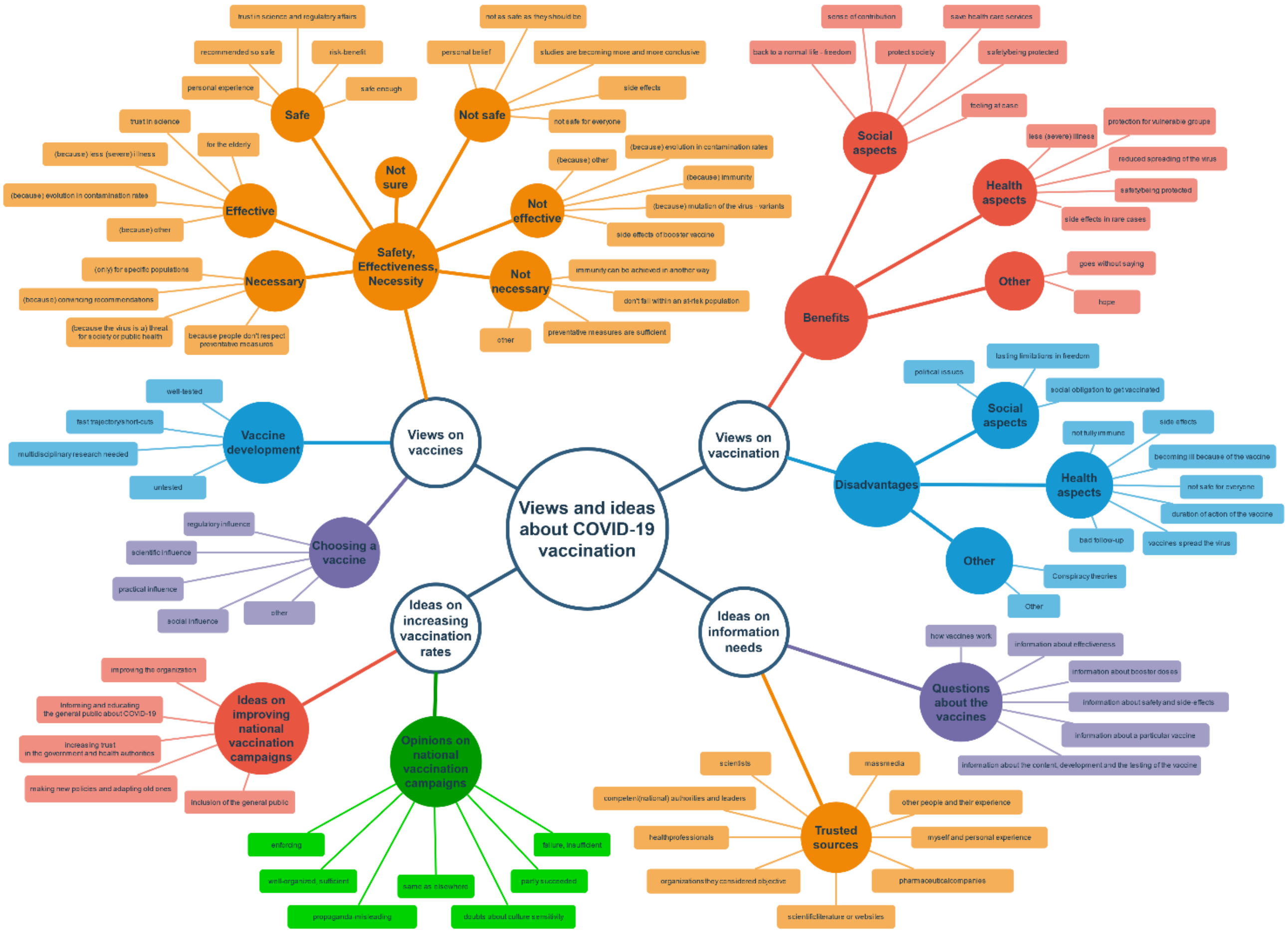

Supplementary File S3. Demographic data of respondents whose quotes are used to illustrate the qualitative data.

#ID	Quote	Country	Sex	Age	Vaccinated for COVID-19?
R1	*"I'm too young to suffer from the side effects of the available vaccines, for a disease/ virus which my body can fight on its own"*	Croatia	Male	27	No
R2	*"Explanation about how it was done so rapidly"*	Slovenia	Female	24	Yes
R3	*"Statistical data on effectiveness"*	Macedonia	Male	59	Yes
R4	*"Tired of COVID-19 information"*	Belgium	Prefer not to say	32	No
R5	*"I think that he who seeks, finds. There is no question at the moment that I have not had an answer to"*	Slovenia	Female	43	No
R6	"safe enough"	Latvia	Female	50	Yes
R7	*"Because I assume that the people who developed these vaccines know what they are doing. Studied for years for this and would not just put something on the market"*	Belgium	Male	23	Yes
R8	*"Without side effects after vaccination"*	Germany	Male	23	Yes
R9	*" to protected myself and others around me"*	Albania	Female	22	Yes
R10	*"all vaccines, even if they have been the most important discovery of medicine in the last 300 years, could, however, in some very rare cases develop adverse reactions, even severe ones"*	Italy	Male	74	Yes
R11	*"vector vaccines are not as safe as they should be"*	Slovenia	Female	34	Yes
R12	*"MRNA vaccines playing with fire despite the pseudo certainties of Pfizer"*	Belgium	Male	82	Yes
R13	*"still a bit afraid of symptoms that would appear later on (higher risk for blood clots or heart attacks)"*	Croatia	Female	24	No
R14	*"even scientists cannot know if the vaccines will be completely safe in the long term"*	Belgium	Female	22	No
R15	*"the COVID-19 vaccines help older people get over the disease more easily"*	Albania	Female	18	No
R16	*"some vaccines are more effective than others depending on who produces them"*	Italy	Male	74	Yes

R17	*"does not help against spreading the disease"*	Germany	Male	28	Yes
R18	*"in preventing the propagation of the virus"*	Slovenia	Female	40	Yes
R19	*"still had COVID-19 after 2 doses"*	Macedonia	Male	27	Yes
R20	*"knew people with three doses admitted to serious intensive care"*	Italy	Female	42	Yes
R21	*"do not seem effective for the current variants"*	Macedonia	Female	27	No
R22	*"not equally effective for everyone (variability) "*	Slovenia	Female	22	Yes
R23	*"people vaccinated with 2 doses were dying"*	Macedonia	Female	41	Yes
R24	*"compromises immunity"*	Macedonia	Female	55	Yes
R25	*"they are effective for their purpose, but those effects are harmful for us"*	Macedonia	Female	21	No
R26	*"In order to prevent infections from occurring in pockets of the unvaccinated population around the world that would lead to subsequent mutations (variants) of the virus with consequences that cannot yet be predicted by current scientific knowledge"*	Italy	Male	74	Yes
R27	*"via herd immunity, particularly immunocompromised people who may not be vaccinated"*	Germany	Female	23	Yes
R28	*"I believe that acquired immunity is better, but i do agree that vaccines are a far better option especially for immunocompromised people"*	Macedonia	Female	22	Yes
R29	*"I have never in my life been vaccinated and in very good health"*	Belgium	Female	50	No
R30	*"I am young and healthy; chances are slim that I would be seriously ill from COVID-19. Therefore, I am not willing to take the risks that come with being vaccinated"*	Belgium	Female	23	No
R31	*"Available vaccines have proven to be ineffective in the long term, and therefore unnecessary"*	Croatia	Male	43	No
R32	*"It's less risky to get COVID than to get vaccinated"*	Italy	Female	56	Yes
R33	*"enough people with acquired immunity"*	Slovenia	Male	57	Yes
R34	*"protective measures are sufficient, if everyone sticks to them"*	Croatia	Female	35	Yes
R35	*"we will live with corona just as we live with the flu"*	Slovenia	Female	24	Yes

Supplementary File S4. Logistic regression to test associations between participants' views on the vaccine's safety, effectiveness, and necessity and demographic factors

Supplementary Table S1. Regression analysis results for the statement 'COVID-19 vaccines are safe' (R square = 0.411, Adjusted R square = 0.389, *P ≤ 0.05).

Model	Unstandardized Coefficients		Standardized Coefficients	t	*P* value
	B	Std. Error	Beta		
(Constant)	-1.097	0.629		-1.745	0.08
Sex: (F=1 M=2)	0.087	0.099	0.028	0.886	0.38
Age: __ years	-0.007	0.004	-0.066	-1.666	0.096
Highest education level (Elementary=1 Doctorate=5)	0.002	0.056	0.001	0.036	0.97
Are you living alone? (No=1, Yes=2)	0.078	0.136	0.019	0.573	0.57
Do you have children under 18 years old? (No=1, Yes=2)	0.127	0.108	0.039	1.170	0.24
Do you personally know anyone who has been hospitalised for COVID-19? (No=1, Yes=2)	-0.059	0.106	-0.018	-0.556	0.58
Have you had all the regular vaccines on the vaccination schedule in your country? (No=1, Yes=2)	0.005	0.261	0.001	0.018	0.99
Have you been vaccinated for COVID-19? (No=1, Yes=2)	1.380	0.128	0.358	10.766	<.001*
I am well informed about the different COVID-19 vaccines used in my country's vaccination program. (Very much agree=5)	0.505	0.034	0.472	14.927	<.001*
Which country do you live in=Albania	-0.185	0.199	-0.037	-0.929	0.35
Which country do you live in=Another country	0.507	0.257	0.067	1.975	0.049*
Which country do you live in =Belgium	-0.170	0.190	-0.035	-0.897	0.37
Which country do you live in =Croatia	0.220	0.166	0.050	1.326	0.19
Which country do you live in =Germany	0.171	0.192	0.033	0.894	0.37
Which country do you live in =Italy	0.056	0.201	0.010	0.280	0.78
Which country do you live in =Latvia	-0.024	0.192	-0.004	-0.125	0.90
Which country do you live in =Macedonia	-0.116	0.173	-0.025	-0.672	0.50
Area of living=Other	0.084	0.515	0.005	0.164	0.87
Area of living=Rural	0.036	0.140	0.009	0.259	0.80
Area of living=Suburban	-0.166	0.123	-0.046	-1.348	0.18
Employment: Studying	0.131	0.182	0.026	0.719	0.47
Employment: Prefer not to say	-0.543	0.359	-0.048	-1.514	0.13
Employment: Other	0.098	0.150	0.024	0.656	0.51

Supplementary Table S2. Regression analysis results for the statement 'COVID-19 vaccines are effective' (R square = 0.370, Adjusted R square = 0.346, *$P \leq 0.05$).

Model	Unstandardized Coefficients		Standardized Coefficients	t	Sig.
	B	Std. Error	Beta		
(Constant)	-0.526	0.624		-0.844	0.40
Sex: (F=1 M=2)	0.064	0.099	0.022	0.647	0.52
Age: __ years	-0.009	0.004	-0.092	-2.196	0.028*
Highest education level (Elementary=1 Doctorate=5)	0.047	0.057	0.030	0.836	0.40
Are you living alone? (No=1, Yes=2)	-0.046	0.136	-0.012	-0.338	0.74
Do you have children under 18 years old? (No=1, Yes=2)	0.036	0.109	0.012	0.330	0.74
Do you personally know anyone who has been hospitalised for COVID-19? (No=1, Yes=2)	0.040	0.107	0.013	0.371	0.71
Have you had all the regular vaccines on the vaccination schedule in your country? (No=1, Yes=2)	-0.100	0.259	-0.013	-0.387	0.70
Have you been vaccinated for COVID-19? (No=1, Yes=2)	1.250	0.129	0.340	9.672	<0.001*
I am well informed about the different COVID-19 vaccines used in my country's vaccination program. (Very much agree=5)	0.447	0.034	0.437	13.072	<0.001*
Which country do you live in=Albania	0.334	0.202	0.071	1.655	0.098
Which country do you live in=Another country	0.604	0.259	0.083	2.332	0.020*
Which country do you live in =Belgium	-0.161	0.193	-0.034	-0.836	0.40
Which country do you live in =Croatia	0.195	0.170	0.046	1.145	0.25
Which country do you live in =Germany	0.442	0.192	0.090	2.298	0.022*
Which country do you live in =Italy	0.345	0.196	0.070	1.760	0.079
Which country do you live in =Latvia	0.269	0.199	0.051	1.350	0.18
Which country do you live in =Macedonia	0.092	0.177	0.021	0.522	0.60
Area of living=Other	-0.991	0.509	-0.066	-1.944	0.052
Area of living=Rural	-0.098	0.146	-0.026	-.676	0.50
Area of living=Suburban	0.024	0.125	0.007	0.194	0.85
Employment: Studying	-0.238	0.183	-0.050	-1.298	0.20
Employment: Prefer not to say	-0.646	0.355	-0.061	-1.818	0.069
Employment: Other	0.230	0.147	0.059	1.571	0.12

Supplementary Table S3. Regression analysis results for the statement 'COVID-19 vaccines are necessary' (R square = 0.378, Adjusted R square = 0.354, *$P \leq 0.05$).

Model	Unstandardized Coefficients		Standardized Coefficients	t	Sig.
	B	Std. Error	Beta		
(Constant)	-0.534	0.697		-0.765	0.44
Sex: (F=1 M=2)	0.166	0.110	0.050	1.504	0.13
Age: __ years	-0.010	0.005	-0.090	-2.145	0.032*
Highest education level (Elementary=1 Doctorate=5)	0.060	0.063	0.034	0.951	0.34
Are you living alone? (No=1, Yes=2)	-0.027	0.150	-0.006	-0.181	0.86
Do you have children under 18 years old? (No=1, Yes=2)	-0.161	0.123	-0.046	-1.302	0.19
Do you personally know anyone who has been hospitalised for COVID-19? (No=1, Yes=2)	-0.026	0.120	-0.008	-0.221	0.83
Have you had all the regular vaccines on the vaccination schedule in your country? (No=1, Yes=2)	-0.185	0.289	-0.021	-0.638	0.52
Have you been vaccinated for COVID-19? (No=1, Yes=2)	1.391	0.146	0.330	9.512	<.001*
I am well informed about the different COVID-19 vaccines used in my country's vaccination program. (Very much agree=5)	0.528	0.038	0.462	13.914	<.001*
Which country do you live in=Albania	0.060	0.225	0.011	0.268	0.80
Which country do you live in=Another country	0.431	0.290	0.052	1.486	0.14
Which country do you live in =Belgium	-0.132	0.212	-0.025	-0.623	0.53
Which country do you live in =Croatia	0.130	0.192	0.027	0.679	0.50
Which country do you live in =Germany	0.481	0.217	0.085	2.219	0.027*
Which country do you live in =Italy	0.416	0.222	0.073	1.871	0.062
Which country do you live in =Latvia	0.134	0.213	0.024	0.630	0.53
Which country do you live in =Macedonia	-0.078	0.197	-0.016	-0.398	0.69
Area of living=Other	-0.660	0.569	-0.039	-1.161	0.25
Area of living=Rural	0.009	0.158	0.002	0.059	0.95
Area of living=Suburban	-0.088	0.138	-0.023	-0.639	0.52
Employment: Studying	-0.186	0.202	-0.035	-0.919	0.36
Employment: Prefer not to say	-1.368	0.415	-0.110	-3.297	0.001*
Employment: Other	0.173	0.167	0.039	1.036	0.301
